# Are There Gender Differences in Performance in Competition in China? An Empirical Investigation

**DOI:** 10.3390/bs15070938

**Published:** 2025-07-11

**Authors:** Gerald Wu, Nikita Nikita, Grace Lordan

**Affiliations:** London School of Economics and Political Science, Houghton St., London WC2A 2AE, UK; nikita1@lse.ac.uk (N.N.); g.lordan@lse.ac.uk (G.L.)

**Keywords:** competition, China, gender differences, labour market, Global South, performance in competition

## Abstract

Evidence from the lab suggests that women perform less well than men under competitive conditions, but the majority of this evidence relates to Western countries. Our study explores gender differences in performance in competitive environments among Chinese individuals. Using a five-round online experimental design, we recruited undergraduate and postgraduate students from a Shanghai university. Participants completed a series of word memory games under varying incentive schemes, including baseline, piece-rate, risk-based, and tournament-style competition. The results of this study suggest that there are no differences in performance under competitive conditions between Chinese men and women. However, women perform slightly better than men when the element of risk is added in a competitive environment. This study underscores the importance of examining cultural nuances when evaluating gender dynamics in competition and contributes to a more comprehensive understanding of these dynamics in the Chinese context.

## 1. Introduction

In the last fifteen years, a rich body of experiments have shown that women often perform less well under competitive conditions than men and tend to avoid competition (Gneezy et al., 2003; Niederle & Vesterlund, 2007; Vandegrift & Yavas, 2009; Ors et al., 2013; Flory et al., 2018). These studies, however, predominantly examine participants from Western, educated, industrialised, rich, developed (WEIRD) societies (Henrich et al., 2010). Given the dearth of evidence outside WEIRD contexts, we ask whether similar gender differences in competitive performance appear in a non-Western setting, namely China.

Studies in diverse cultural contexts do suggest that gender gaps in competitiveness are not universal. For example, Gneezy et al. (2009) found that Maasai women (from a patriarchal society) were less competitive than men, while Khasi women (from a matrilineal society) were more competitive than men. In China, evidence is limited: Carlsson et al. (2020)—in one of the few experiments on this question—reported that Chinese women performed equally well or better than men under competitive pay conditions. These findings underscore that culture may shape competitive behaviour by gender, highlighting the importance of extending research beyond WEIRD samples.

To test for gender differences in performance in competition within China, we designed an online experiment modelled after Gneezy et al. (2003) and Niederle and Vesterlund (2007). We recruited students from a large business school in Shanghai to participate in five rounds of a word-memory game under varying incentive schemes (baseline, piece-rate, risk-added, and tournament competition). This design allowed us to observe whether men and women respond differently to each incentive condition.

Drawing on the existing literature and the cultural context of China (detailed in Section 2), we advance four hypotheses. First, we hypothesise that women and men will perform equally under non-incentivised baseline conditions, reflecting similar innate abilities (H1). Second, we expect that women will perform worse than men under competitive incentive structures, consistent with findings from WEIRD settings (H2). Third, we propose that individuals with high confidence levels will perform better across all conditions, although this effect may differ by gender (H3). Finally, we hypothesise that participants with higher career aspirations will exhibit superior performance, particularly under incentivised settings (H4).

Our results indicate that, overall, women showed no significant performance differences compared to men in incentivised games, suggesting participants in our study did not notably adjust their behaviour to the incentives. This aligns with similar findings in individuals from Taipei (Booth et al., 2019) and Han (Zhang, 2019) backgrounds, demonstrating no discernible gender-based variations in competitiveness. Notably, women outperformed men in environments with added risk, indicating potential gender differences in incentive responses. This is also in line with experimental findings by Carlsson et al. (2020) for China, who found that, in a task introducing a risk element with payments tied to others’ performance, women outperformed men. This contrasts with many Western studies, which have reported women performing less favourably than men in competitive settings (Niederle & Vesterlund, 2011).

Our study, contributing to the literature on gender performance in competition (Azmat & Petrongolo, 2014; Gneezy et al., 2003), aligns with broader research exploring explanations for labour market outcomes and gender pay gaps. While prior studies propose that women’s lower performance in competition contributes to the gender gap (Gneezy et al., 2003; Niederle & Vesterlund, 2007), our findings challenge this notion. Despite the pronounced gender pay gap and barriers faced by women in China (Ding, 2022), our results suggest that differences in competitive ability may not be the primary driver of these disparities.

In summary, this study fills a gap by providing evidence from a non-Western context on gender and competition. The remainder of the paper is structured as follows. Section 2 reviews the relevant literature. Section 3 details our experimental methodology and design. Section 4 presents the results. Section 5 discusses the findings and their implications, and Section 6 concludes the paper.

## 2. Literature Review

### 2.1. Overview of Cultural Influences on Performance in Competition

Chinese culture, shaped by Confucian, Taoist, and other indigenous values, emphasises collectivism, social harmony, and respect for hierarchy. These cultural dimensions can influence competitive behaviours. For instance, a strong collectivist orientation may discourage overt competition to preserve group harmony. In line with this, Chinese women have been found to endorse collectivist values more than men (Ralston et al., 1999), potentially making them less inclined to compete, similar to patterns observed among women in Western studies. At the same time, concepts like “saving face”—maintaining one’s social image—might make individuals of either gender cautious about aggressive competition for fear of public failure. The net effect of these values is ambiguous: on one hand, cultural norms may dampen competitiveness (especially for women seeking harmony), but on the other, Chinese cultural thought also allows that cooperation and competition can coexist (Peng & Nisbett, 1999; Chen et al., 2011). Reflecting this complexity, empirical evidence on gender and competitiveness in China has been mixed. Booth et al. (2019) and Zhang (2019), for example, found that gender differences in competitiveness varied across Chinese ethnic and regional groups, whereas Carlsson et al. (2020) observed that Han Chinese women actually performed slightly better than men in competitive environments. These studies suggest that cultural context can modulate gender gaps in competitive behaviour, making it difficult to predict a priori whether Chinese women will behave like their Western counterparts in competitive settings.

Traditional gender role norms and China’s historically high power-distance society could further impact competitive performance. Confucian traditions have long espoused a subservient role for women, encapsulated by the doctrine of “three obediences” (women’s duty to defer to father, then husband, then son). Such norms may have conditioned women to be less assertive and less willing to compete, even among peers. Consistent with this possibility, Zhang (2019) found that women’s inclination to compete was lower in a traditionally patriarchal subgroup (the Yi people) but not in a more gender-equal subgroup (Han Chinese). This indicates that where strong patriarchal norms persist, women may be less likely to fully exert themselves competitively. Overall, the interplay of collectivist tendencies, face-saving concerns, and hierarchical gender norms in China creates an uncertain theoretical expectation for our study. Chinese cultural values could either suppress gender differences in competition (if both men and women avoid competition) or accentuate them (if women are especially discouraged from competing). This uncertainty motivates our empirical investigation.

### 2.2. The Relevance of Exploring Gender Differences in Performance in Competition in China

The importance of considering whether there are gender differences in performance under competition for a population from China goes beyond simply being a non-WEIRD context. China is one of the fastest-growing economies in the world, with a GDP of USD 14.4 trillion in 2019, making up 16.38% of the global economy. In 2020, 124 Chinese companies with a combined revenue of USD 8.3 trillion appeared on the Fortune Global 500 list, representing nearly a quarter of the USD 33.3 trillion in revenue generated by all 500 companies (China Power Team, 2021). In addition, many WEIRD countries recruit talent from China for professional services, including senior positions. In the United States, there are 2.1 million individuals of Chinese origin in the workforce (Chui et al., 2020), with 60% of Chinese immigrants engaged in management, business, science, and arts roles in 2021 (Rosenbloom & Batalova, 2023). Similarly, in the United Kingdom, 12.8% of individuals from the Chinese ethnic group held positions in ‘higher managerial, administrative, and professional occupations’ (UK Government, 2020).

Finally, China has a notable pay gap between men and women, as the average monthly salary of Chinese women in the workplace is 12% lower than that of men (Ding, 2022). The majority of women remain concentrated in gender stereotypical occupations that tend to involve less risk and lower visibility and are likely to have little authority and lower pay than those of their men counterparts (Adler, 1993; Ohlott et al., 1994; Van Velsor & Hughes, 1990; Westwood & Leung, 1999). Therefore, understanding differences in performance in competition by gender for a Chinese population helps gain an understanding of whether differences in competitive performance are an important ingredient in explaining the occupational choices of men and women in this country, along with the gender pay gap.

## 3. Methods

### 3.1. Participants

An online experiment was conducted in September 2019 with students from the School of Economics and Finance at Shanghai International Studies University (SISU), China. We focused on a single large business school to ensure participants had comparable academic backgrounds, thereby reducing the likelihood that ability differences would drive performance differences in the task we set (we formally test for any baseline ability gap in the first round). While this elite student sample does not represent the general Chinese population, it does capture a cohort likely to become future business leaders, allowing us to investigate gender competitiveness in a high-achieving segment of China’s youth.

Participants were recruited via an official invitation sent by the Dean of the school through “WeChat,” a popular messaging app. Using a respected authority figure as the messenger proved effective: we obtained 480 responses, a high response rate (~35.8%) that may reflect a messenger effect whereby students were especially inclined to respond to the Dean’s request. All participants provided informed consent electronically before beginning the survey. Only current SISU students could participate (verification was required within the survey). Individuals who either declined consent (n = 59), were non-students (n = 12), or did not report their gender (n = 83) were filtered out. Our final sample for analysis comprised 326 participants (239 women and 87 men). This gender breakdown (73% female) reflects the fact that the business school’s student body is largely female (approximately 70% women).

We determined our target sample size through power calculations based on Gneezy et al. (2003). Given the effect sizes they observed between men and women (~9–11 points with SD~5.65) and a desired power of 0.80 at α = 0.05, we estimated needing roughly 446 participants (~223 per gender). Our achieved sample of 326, while below this target, is comparable to or larger than samples in many influential experimental studies on gender and competition. For example, Niederle and Vesterlund (2007) detected a significant gender gap in tournament entry with only 80 participants, and Gneezy et al. (2009) found culture-by-gender effects with approximately 75 subjects per group. Thus, our N = 326 provides substantial statistical power in the context of this literature and was deemed sufficient for analysis.

All participants completed the study online. Recent research indicates that online experiments can yield results as valid as traditional lab experiments. We followed the general experimental protocol of Gneezy et al. (2003), adapting it to an online survey format. The task instructions and interface were provided in both English and Chinese to eliminate language barriers. A rigorous back-translation procedure was used: the English survey was translated to Chinese, then independently back-translated to English to ensure accuracy. Participants received detailed information about the study’s purpose, procedures, and time requirements, and then confirmed consent by checking an agreement box (those who declined were exited from the survey). As a modest incentive for participation, we offered a Tmall.com shopping voucher upon completion, with the exact amount tied to performance (see Section 3.2). Approximately 78% of participants chose to claim this reward, with similar take-up rates and average payouts for women and men.

### 3.2. Reward Structure

To test the impact of different incentive schemes on performance, a modest incentive reward in the form of a T-Mall (天猫) gift voucher for Tmall.com was provided to participants. Tmall.com, similar to Amazon, is a Chinese-language website for local Chinese and international businesses to sell goods to consumers in mainland China, Hong Kong, Macau, and Taiwan. Participants who achieved a total score below 20 points received CNY 20 (GBP 2.28), between 21 to 30 points received CNY 30 (GBP 3.43) and CNY 50 (GBP 5.71) for scores above 31 points.

To help with the distribution of the reward, participants were required to provide their name and email address to collect their award. They were informed that their name and email address would only be used for the distribution of the reward and no other purposes, the information would be destroyed once rewards were distributed, and anonymised data would be retained for statistical analysis.

Overall, 78% (n = 254) of participants were interested in claiming the rewards. Of the 254, 72.83% (n = 185) were women and 27.17% (n = 69) were men. The average payout was CNY 23.78 (approximately GBP 2.71). The average payout between men and women was similar, where women received CNY 23.78, and men received CNY23.77.

This research did not receive any specific grant from funding agencies in the public, commercial, or not-for-profit sectors.

### 3.3. Experiment Design

The online survey required participants to complete five rounds of word memory games developed by the authors to examine gender differences in performance under different incentive structures. Specifically, for each round, 20 words were displayed for 15 s, and participants were required to memorise as many words as possible. Then, on the next screen, 24 words appeared, and participants had 20 s to find the words that appeared in the previous screen. Out of the 24 words, only 12 matched the previous 20 words, making the possibility of having 12 as the highest number of correct answers. The format of each round was the same; the only difference was the incentive structure. By having only one change between rounds, we wanted to learn how it modifies the behaviour of the participants. Specifically, each participant faced five rounds of the game with the incentive structure as follows.

#### 3.3.1. Game 1 (G1) Baseline

Participants’ performance was based only on their inherent ability and effort.

No incentives were provided in this round. This round served to establish whether there were gender differences in ability with respect to the task in this study. Furthermore, it provides a baseline to measure innate performance and effort.

#### 3.3.2. Game 2 (G2) Piece-Rate

Participants received a piece-rate incentive for correct answers. For each word correctly identified, one point was earned; points accumulated counted toward the final score and thus the reward tier. This incentive was a reward based on one’s own performance, independent of others.

#### 3.3.3. Game 3 (G3) Piece-Rate + Risk

A risk element was added to the piece-rate incentive. In G3, participants gained two points per correct answer but lost two points for each incorrect answer. This negative scoring for errors introduced uncertainty and risk, potentially discouraging guessing. Prior research suggests women tend to be more risk-averse (Byrnes et al., 1999; Paserman, 2007); thus, G3 tested if adding risk differentially impacts men’s vs. women’s performance by perhaps causing risk-averse participants to hold back.

#### 3.3.4. Game 4 (G4) Tournament + Risk

A competitive tournament incentive was introduced, combined with a risk component. In G4, a participant’s points from this round only counted toward their final score if the participant placed in the top 10 scores among all participants. Within this tournament condition, correct answers earned five points each, and incorrect answers incurred a five-point penalty. The five-point deduction was maintained to prevent participants from simply selecting all options to guarantee a high score. (Any participant ending with negative total points still received the minimum CNY 20 voucher to ensure no one was “penalised” monetarily for participating).

#### 3.3.5. Game 5 (G5) Return to Baseline

All incentives were removed again in the final round. G5 was identical to G1 (unpaid) but occurred after participants had practised with the task. Comparing G5 to G1 allows us to see if any learning or fatigue occurred over the session and whether men or women improved differently with practice.

At the end of the survey, participants were asked to provide demographics (gender, age, education, etc.), their career aspirations, and to self-evaluate their performance compared to others.

The format of the tasks is closely aligned with the design of Gneezy et al. (2003) in the following ways. First, participants were required to complete the same task under different incentive schemes to allow the impact on performance to be measured. Second, the word memory games were selected as there is no evidence to suggest that there would be a difference in ability between men and women completing the task. We will check for differences in the results found in the first and the last round of the game. Third, the task is relatively easy to explain and conducted via an online survey.

### 3.4. Measures

#### 3.4.1. Dependent Variables

Similar to the approach taken by Gneezy et al. (2003), in evaluating participants’ performance, the key metric is the participants’ scores, which become the base for creating dependent variables. We have three outcome variables: (1) standardised score, (2) top quintile status (a binary variable = 1 if a person performs in the top quintile and zero otherwise) and (3) bottom quintile status (a binary variable = 1 if a person performs in the bottom quintile and zero otherwise). Together, these outcomes enable us to explore if men and women differ in performance in response to our various incentives in terms of their average performance, and also the likelihood of being exceptional or an underperformer.

Table 1 shows the average score of participants in each game as well as the proportion of top quintile and bottom quintile for women and men, respectively. While men participants, on average, seem to have marginally higher scores in G1 and G2, their women counterparts had a higher average score in G3, G4 and G5. In terms of quintile distribution, as depicted in Table 1, in G1, both men and women have equal representation in the bottom quintile, while in the top quintile, men have a greater share than women in G1, G2, and G5. Conversely, G3 and G4 exhibit a shift with a higher proportion of women in the top quintile and a higher proportion of men in the bottom quintile. Furthermore, G5 once again displays a higher proportion of men in the bottom quintile than women.

Through the series of five-game sets, each featuring varying levels of incentives, this study introduces five experimental groups to examine the impact of incentives on performance levels. By analysing these quintile-based variables alongside the standardised score, we aim to gain a comprehensive perspective on participants’ performance within the context of the game’s diverse incentive structure.

#### 3.4.2. Independent Variables

There are two primary independent variables in this study: first, the gender of the participants, where 0 was assigned to participants who were men and 1 to women. All gender status information was based on self-report. As seen from Table 1, of the 326 participants, 73.31% (n = 239) were women, and 26.69% (n = 87) were men. This is closely representative of the current gender breakdown of the school’s student population, namely 70% women and 30% men.

The second is the set of variables that represent the different incentive schemes. That is, we created a set of variables that are assigned = 1 if an individual was engaged in G1, G2, G3, G4 or G5 when an outcome is being captured, and 0 otherwise.

#### 3.4.3. Control Variables

In line with Gneezy et al. (2009), we controlled for key demographics of the participants such as age, current education level, and area of study. We captured this information at the end of the experiment (see Table 1).

Studies have found that competitiveness preferences may influence career decisions (Dittrich et al., 2014; Leibbrandt & List, 2014; Buser et al., 2014), and therefore, this has been included as one of the controlled variables as career aspirations. More specifically, we measured preferences for long-term career aspirations through career categories based on the International Standard Classification of Occupations by the International Labour Organization (International Labour Office 2012). These categories ranged from managers, professionals, armed forces officers, sales workers, and clerical workers to agricultural-based roles, technicians, and manufacturing workers. To the ILO occupation classifications, three additional categories were added to make the categorisation well-rounded: senior executives (e.g., Senior Officials, Managing Directors, and Chief Executives), home keepers and self-employed. For the purpose of this study, those with career aspirations of being a senior executive are further categorised as participants with ‘high career aspirations’.

In the data analysis, we treat high career aspirations as a binary variable identifying participants with high career aspirations and those who do not (participants who chose the rest of the career categories), and considering whether this impacts the response to incentives. In Table 1, we can see that 46.4% (n = 151) of the sample have high career aspirations of becoming senior executives. There is a higher proportion of men (54.7%) who would like to be senior executives when compared to women (43.3%).

Lastly, we control for the participants’ confidence levels following Niederle and Vesterlund (2007). To assess the subject’s confidence level, upon completing the five rounds of the task, participants were asked to self-evaluate their overall performance compared to all other participants. The participants were asked to self-estimate their overall score in one of the following five categories: (1) “50% of participants performed better than you”, (2) “75% of participants performed better than you”, (3) “Your overall score is in the Top 1%”, (4) “Your overall score is in the Top 10%”, and (5) “Your overall score is in the Top 25%”. For ease of interpretation, in the study, we further categorise those participants who self-estimated their overall score to be in the top 1%, 10% or top 25% as participants with a ‘high-confidence level’. Those who either stated that 50% of participants performed better than them or 75% of the participants performed better than them have been treated as participants with a ‘low-confidence level’.

In the data analysis, we treat confidence level as a binary variable identifying participants with high-confidence levels and low-confidence levels, and considering if this caused variation in the response to incentives. In Table 1, we see that only 27.1% (n = 88) of participants showed a high confidence level, i.e., self-estimated their overall score to be in the top 1% or 10% or top 25%. Overall, 72.9% (n = 238) of the participants showed low confidence as they either stated that 50% of participants performed better than them or 75% of the participants performed better than them.

While participants received no feedback on other participants’ performance, they did receive feedback on their own performance after each game in accord with Niederle and Vesterlund (2007).

#### 3.4.4. Hypotheses

As we discussed in the introduction, we consider four main research questions in this study.

**Hypothesis 1.**  *The performance of women is expected to be equal to the performance of men.*

To examine this hypothesis, we estimate:

(1) $$Y_{i}=\;\beta_{0}+\;\beta_{1}{Woman}_{i}+\gamma X+\;\varepsilon_{i}$$

In Equation (1), ‘$Y_{i}$’ refers to the outcome variables (correct answers and standardised score) for individual ‘*i*’, ‘$\beta_{0}$’ is the intercept, ‘${Woman}_{i}$’ is a dummy variable identifying if a participant is a woman (=1), and = 0 if the participant is a man. ‘X’ is a vector of covariates, and ‘*ε*’ captures the error term. We will confirm hypothesis 1 if $\beta_{1}$ is centred around zero and not significant. We estimated this model twice using only the data from G1 and G5, the two rounds of our game where there were no incentives. We note that the only difference between G1 and G5 is that by the time participants are engaging in G5 they have had time to practice the game, so we expect their ability to be higher. This implies that we predict that overall scores are higher, but do not differ between men and women.

**Hypothesis 2.**  *The effect of gender and incentives will interact with each other such that women are expected to perform lower than men in games with incentives.*

To examine this hypothesis, we estimate:

(2) $$Y_{ij}=\;\beta_{0}+\;\beta_{1}{Woman}_{i}+\beta_{2}\;{Game}_{j}+\delta \left({Woman}_{i}\times {Game}_{j}\right)+\gamma X+\;\varepsilon_{ij}$$

‘${Game}_{j}$’ is a dummy variable, for each game ‘*j*’, assigned equal to 1 when the participant faces a particular game (namely G1, G2, G3, G4 or G5) and 0 otherwise. Recall that each game offers the participant a distinct incentive structure. Therefore, ‘${Woman}_{i}\times {Game}_{j}$’ is a vector of interaction terms, whose associated vector coefficients to be estimated explicitly, tests whether men and women respond differently to the incentives offered in each game.

As described in hypothesis 1, we may expect men and women to perform the same in G1 and G5. In contrast, G2, G3, and G4 present distinct motivators for participants. Specifically, in G2, participants receive a piece-rate incentive for each accurate response. In G3, an element of uncertainty is introduced, where participants earn two points for each correct answer but face a deduction of two points for each incorrect answer. In G5, the stakes are heightened further, with participants gaining five points for every correct response but losing five points for each incorrect response.

We may expect women to perform worse than men if they have a lower tendency to compete in line with previous literature from China (Booth et al., 2019; Zhang, 2019) and WEIRD countries (Gneezy et al., 2003; Niederle & Vesterlund, 2007; Vandegrift & Yavas, 2009; Ors et al., 2013; Flory et al., 2018). This would imply negative and significant coefficients on the ‘${Woman}_{i}\times {Game}_{j}$’ that relate to G2, G3 and G4. However, as we discussed in Section 2, it is not clear whether women will respond differently in games G2, G3 and G4.

**Hypothesis 3.**  *The performance of participants with high confidence levels is expected to be higher than the performance of participants with low confidence levels.*

That is, we estimate:

(3) $$\begin{array}{c} Y_{ij}=\beta_{0}+\beta_{1}{Woman}_{i}+\beta_{2}\;{Game}_{j}+\beta_{3}\;\left({Confidence}_{i}\right)+\delta \left({Woman}_{i}\times {Game}_{j}\right)\\ \;\;\;\;\;\;\;\;+\;l\left({Woman}_{i}\times {Confidence}_{i}\right)+\;\theta \left({Confidence}_{i}\times {Game}_{j}\right)\\ \;\;\;\;\;\;\;\;+\;\nu \left({Woman}_{i}\times {Game}_{i}\times {Confidence}_{i}\right)+\gamma X+\;\varepsilon_{ij} \end{array}$$

In Equation (3), ‘${Confidence}_{i}$’ is a dummy variable identifying participants with high-confidence level (1) versus low-confidence level (0). ‘${Woman}_{i}\times {Confidence}_{i}$’ is the interaction term whose coefficient tests whether the impact of participants’ confidence levels on the dependent variable differs between men and women. ‘${Confidence}_{i}\times {Game}_{j}$’ interaction term investigates whether the relationship between the participants’ confidence level and the dependent variable varies depending on the game type. ‘${Woman}_{i}\times {Game}_{i}\times {Confidence}_{i}$’ interaction investigates whether the combined influence of game type, confidence level and gender has a distinct effect on the dependent variable.

**Hypothesis 4.**  *The performance of participants with high career aspirations is expected to be higher than the performance of participants with low career aspirations.*

That is, we estimate:

(4) $$Y_{ij}=\beta_{0}+\beta_{1}{Woman}_{i}+\beta_{2}\;{Game}_{j}+\beta_{3}+\delta \left({Woman}_{i}\times {Game}_{j}\right)+\theta \left({CareerAsp}_{i}\times {Game}_{j}\right)\;\;+\nu \left({Woman}_{i}\times {Game}_{j}\times {CareerAsp}_{i}\right)+\gamma X+\;\varepsilon_{ij}$$

‘${CareerAsp}_{i}$’ is a dummy variable identifying participants with high aspirations (1) and low aspirations (0). ‘${Woman}_{i}\times {CareerAsp}_{i}$’ is the interaction term that explores whether the relationship between participants’ career aspirations and the dependent variable differs based on gender. ‘${CareerAsp}_{i}\times {Game}_{j}$’ interaction term explores whether the relationship between participants’ career aspirations and the dependent variable changes depending on the game type. ‘${Woman}_{i}\times {Game}_{i}\times {CareerAsp}_{i}$’ aims to determine whether the combined effects of game type and career aspirations on the dependent variable differ between men and women.

In Equations (2)–(4) above, ‘$Y_{ij}$’ is an outcome variable that links to the performance of participant ‘*i*’ in game ‘*j*’. When considering each individual score, we consider three different dependent variables for each measure. The first is the standardised level of the score attained, the second is a dummy variable that is = 1 if an individual was among the top quintile highest achievers and zero otherwise, and the third is a dummy variable that is = 1 if an individual was among the bottom quintile lowest achievers and zero otherwise.

Initially, we examine all equations without any control variables, followed by a subsequent analysis that incorporates control variables. In addition, we also estimate versions of Equations (2)–(4), which include individual fixed effects. For a comprehensive overview of the variable changes made to the baseline models in Equations (1)–(4) for various testing purposes, please refer to the regression tables in Section 4.

## 4. Results

### 4.1. Hypothesis 1

Table 2 presents the results of multiple regression models assessing the impact of gender on standardised scores and score quintiles in two games: Game 1 (G1) and Game 5 (G5). The table comprises several models with different dependent variables: models (1), (2), and (3) examine standardised scores, top 20% achievers (Top 20), and bottom 20% achievers (Bottom 20) in G1, respectively; models (4), (5), and (6) evaluate the same outcomes for G5.

The analysis in Table 2 reveals no statistically significant gender-based differences in either standardised scores or score quintiles across G1 and G5. Consequently, these findings do not provide sufficient evidence to reject Hypothesis 1, i.e., women and men are expected to perform equally in G1 and G5. This supports the conclusion that there are no inherent differences in ability between genders in executing the task. Therefore, any performance variations observed in Games 2, 3, and 4 can be attributed to the incentive structures rather than baseline ability or differences in learning speed.

### 4.2. Hypothesis 2

Table 3 provides insights into hypothesis 2, which investigates the influence of being a woman versus being a man on standardised scores (models 1, 2, and 3), the likelihood of ranking within the top 20% (models 4, 5, and 6), and the likelihood of ranking within the bottom 20% (models 7, 8, and 9) across different games (G1 to G5). Models 2, 3, 5, 6, 8, and 9 include control variables, while Models 3, 6, and 9 also incorporate individual fixed effects. These models also incorporate an interaction term with a woman dummy and the specific games to understand how gender impacts performance across the various incentives offered with the game.

In column 1, the coefficient for “woman” is not significantly different from zero, indicating on average, women do not perform differently from men in the game. We draw similar conclusions when we consider the estimates documented in columns 3 and 5, which relate to the odds of performing in the top and bottom 20%, respectively. We note that this coefficient becomes more substantive in model 2 when controls are added to the model that considers the standardised scores, indicating that there may be differences between men and women in performance that interact with the observed control variables. We explore this possibility subsequently.

For the game-specific effects, we mostly do not observe any differences in performance across the games for participants, regardless of whether we consider. This suggests that the participants are mostly not responding to the incentives we set. The exception is game G3, where participants receive a piece-rate incentive, along with an element of risk. More specifically, participants receive two points for each correct answer, and two points are deducted for each incorrect answer. Here, the coefficient for “G3” is statistically significant in models 1 to 3. It is −0.39, suggesting that, on average, participants score approximately 0.4 standard deviations lower in Game 3 compared to Game 1. This difference is statistically significant at the 1% level. This suggests that, on average, men in the study shied away from risk, as compared to their performance in G2 when a simple piece rate was added. Women perform slightly better, as depicted by the significant and positive effects on the “Woman × G3” interaction. Overall, our results suggest that women have, on average, standardised scores that are 0.5 standard deviations higher than men’s, and 0.1 standard deviations. Overall, our results suggest that it is men, not women, who shy away from exerting effort when there is a risk element added to the games, with women performing slightly better.

### 4.3. Hypothesis 3

Table 4 provides insights into hypothesis 3, which explores the combined effects of gender, incentives, and our participant’s confidence levels on standardised scores (models 1, 2, and 3) as well as the probability of ranking within the top 20% (models 4, 5, and 6) and the bottom 20% (models 7, 8, and 9) across different games (G1 to G5). Recall that high confidence is a binary variable, taking a value of 1 when a participant self-assesses their overall performance as being within the top 1%, 10%, or 25%. Conversely, it assumes a value of 0 when a participant’s self-assessment indicates that 50% or 75% of the participants outperformed them.

We note in Table 4 that the coefficient on high confidence and the interaction between high confidence and the woman dummy is not identified because of multi-collinearity with the individual fixed effect.

As expected, having high confidence levels improves performance in the standardised scores as well as the probability of being a top or a bottom performer. However, when we consider the probability of being among the top 20% performers, it is noteworthy that the confidence gains are attenuated for women, as illustrated by the negative coefficient between the woman dummy and the high confidence dummy. More specifically, from columns 4 and 5, men with high confidence are 0.39 percentage points more likely to be among the top 20 per cent performers. For women, the same figure is 0.15.

Including high-confidence variables in the regressions explaining variation in standardised scores once again highlights that it is men, rather than women, who perform worse when a risk element is introduced through piece-rate incentives, as seen in the G3 coefficients. Notably, the coefficients associated with G4 are now statistically significant in Table 4. Recall that in the G4, participants face a competitive incentive structure layered onto the risk-based incentives already present in G3; specifically, individual scores depend not only on absolute performance but also on relative performance compared to others. Once confidence is accounted for, Table 4 indicates that men perform worse in G4 relative to both women and the G1 baseline. In contrast, women perform slightly better. Quantitatively, men’s performance in G4 drops by approximately 0.4 standard deviations below the baseline, whereas women’s performance remains roughly equivalent to the baseline level.

We note that the coefficients that relate to the triple interaction effects between each game, the woman dummy, and the high confidence are never significant. This indicates that gender, confidence, and the specific incentive structures of each game do not interact in a way that causes significant differences in performance among the participants. Overall, the findings from Table 4 support Hypothesis 3, which posits that participants with a high confidence level would outperform those with low confidence. Furthermore, the results in Table 4 suggest that it is men rather than women whose performance declines when faced with incentives that concern increased risk and competitiveness.

### 4.4. Hypothesis 4

Table 5 documents our estimates, which explore the relationship between gender, incentives, and career aspirations. Overall, the conclusions in Table 5 mirror those documented in Table 3. Notably, none of the coefficients that relate to the interactions with career aspirations are significant. This implies that we cannot reject the null hypothesis that the performance of participants with high career aspirations is the same as the performance of participants with low career aspirations.

## 5. Discussion

Despite the predominant role of China in the global economy, only three research studies have previously been conducted to explore gender differences in performance among Chinese individuals. Our study analysed, therefore, whether Chinese men and women differ in performance under competitive conditions and varying incentives. We modelled our experiment on well-known designs from the literature and specifically investigated how gender interacted with the incentive schemes, shedding light on competitive behaviour in a Chinese context.

Overall, our findings reveal that women, on average, performed on par with men across the different incentive games. In general, introducing incentives did not cause women to significantly underperform relative to men. This outcome aligns with similar findings from Taipei (Booth et al., 2019) and among Han Chinese individuals (Zhang, 2019), where no discernible gender differences in competitiveness were observed. By contrast, many studies in Western countries have reported that women perform worse or shy away from competition compared to men (Niederle & Vesterlund, 2011). Our divergent result reinforces the notion that competitive behaviours are heavily influenced by cultural context and are not universally generalisable. In a Chinese environment, at least for our educated, young sample, women did not exhibit the same competitive disadvantage documented in the Carlsson West. This suggests that factors such as social norms, upbringing, and educational context in China may encourage women’s performance in competitive situations or at least not inhibit it.

A notable exception in our experiment was the game involving a risk element (Game 3). Here, we observed a clear gender difference: faced with potential penalties for wrong answers, men tended to reduce their effort (or effectiveness), while women’s performance slightly improved relative to baseline. In this risk-added condition, women actually outscored men, indicating that women handled the risk incentive better. This finding is striking because it contradicts the common assumption (derived from earlier literature) that women are more risk-averse and would therefore underperform in uncertain, high-risk environments (Paserman, 2007; Byrnes et al., 1999). In our Chinese sample, men appeared more deterred by the risk than women. Our result closely echoes Carlsson et al. (2020), who likewise found that Chinese women outperformed men when a competitive task introduced a risk of payoff based on others’ performance. Together, these findings suggest that the interaction of gender and risk in competitive settings may be culturally specific. It is possible that Chinese women (especially younger, highly educated ones) are becoming more comfortable with risk in achievement contexts, or that Chinese men in this cohort are relatively more cautious than expected. Evolving societal expectations and gender roles in modern China—for instance, increasing encouragement for women to pursue careers and leadership—might be narrowing or even reversing traditional gender gaps in risk-taking. This is an area ripe for further investigation.

In summary, our study’s outcomes diverge from many WEIRD-based studies where women underperform in competitive contests, and instead resemble findings by Carlsson et al. (2020) and Zhang (2019) in China, which showed little or no female disadvantage (and even a female edge in some cases). This implies that gender gaps in competitive performance may be culture-dependent rather than universal. The prevailing theories developed from Western experiments—for example, that women’s aversion to competition contributes to their lower advancement—may not straightforwardly apply in the Chinese context. Our evidence calls into question the global generality of Western-centric research on gender competitiveness and underscores the importance of testing these phenomena in diverse settings.

We also examined the roles of confidence and career aspirations in performance. Not surprisingly, participants who were more confident in their abilities tended to perform better (higher scores and higher chance of top rankings), which is consistent with broader research linking confidence to improved task performance and persistence. However, we did not find that this effect differed by gender: high confidence boosted women’s and men’s performance similarly, and the interaction between gender and confidence was not statistically significant. In practical terms, a confident woman was just as likely as a confident man to reap performance benefits. We also tested whether having high career aspirations (aiming for top-level jobs) corresponded to better performance, theorising that ambitious individuals might exert more effort in competitive tasks. In our data, career aspirations did not translate into any performance advantage—nor did we detect any significant interactions with gender on this front. It appears that simply wanting a high-powered career did not make participants perform any better in these games once other factors were controlled for. This could be because many factors beyond raw ambition (such as current skills, experience, or situational factors) determine performance in a given task.

Crucially, our findings suggest that women, on average, are just as capable as men in competitive performance, and in some cases (with risk involved) may even outperform men. This indicates that differences in the ability or willingness to compete are unlikely to be the primary driver of the gender pay gap or women’s underrepresentation in senior roles in China. In other words, the explanation for why Chinese women are less prevalent in top positions or earn less than men is probably not that they perform worse when competing. Other factors—such as gender differences in choosing to enter competitions, discriminatory barriers, differences in networking or mentorship, or work–family tradeoffs—might play more significant roles and warrant investigation.

There are several limitations to our study that qualify these conclusions. First, our sample was not representative of the entire Chinese population. Participants were drawn from an elite university’s business school, meaning they were mostly young (18–32 years old), well-educated, and likely quite career-oriented individuals in Shanghai. The competitive behaviours of this group may not generalise to older cohorts or those with different educational backgrounds. In fact, prior research suggests that age can affect competitiveness—older individuals tend to be less willing to engage in competition (e.g., Garratt et al., 2013). It is possible that a middle-aged sample, or one from a more rural area, would show different gender dynamics. Future research should include a broader demographic range to see if the patterns we observed hold true across various segments of Chinese society. Expanding the participant pool beyond students (to working professionals, people from different regions of China, etc.) would enhance the external validity of the findings.

Second, in our experimental design, participants all completed the games in the same fixed order (G1 through G5). This non-randomised sequence means there could be order effects or learning effects that influenced results—for instance, everyone might improve in later games simply due to practice, or fatigue might set in toward the end. While we tried to mitigate learning by including G5 to measure end-of-session baseline ability, it is possible that the sequence itself impacted behaviour in ways unrelated to the incentives. For example, by the time participants reached G4 (tournament), they knew their own performance patterns and may have been more or less motivated. Future studies should randomise the order of incentive conditions for different participants to control for sequence effects and ensure that any performance differences are truly due to incentive structures rather than task order.

Third, our study specifically measured performance under competitive conditions (how well participants performed when placed in competition), but it did not directly measure the willingness to enter competition. Much of the existing literature on gender and competition, including studies in China, focuses on the latter—i.e., whether women opt into competitive environments or prefer non-competitive compensation. Our results contribute a complementary perspective by showing that, once in a competition, women performed as well as men. However, it could be that fewer women than men would choose to compete in the first place, even if their performance would be equal. Thus, an important direction for future research is to examine both selection and performance: do Chinese women and men differ in choosing competitive challenges, and how does that relate to their eventual performance? Combining these dimensions would provide a more complete understanding of gender dynamics in competition. Integrating our performance findings with data on competitive entry would help determine to what extent China’s gender gaps might be due to participation differences versus performance differences.

In summary, our study challenges some assumptions derived from Western-centric research and underscores the importance of cultural nuance when evaluating gender differences in competition. We find that in a Chinese sample, women are every bit as competitive in performance as men, and can even excel in certain competitive scenarios involving risk. These insights contribute to a more comprehensive understanding of gender and competition by adding evidence from the Chinese context. As China’s role in the global economy continues to grow, understanding how gender dynamics play out in its workforce is increasingly important. Our findings suggest that *if* Chinese women are given the opportunity to compete on an equal footing, their performance will match that of men. This has encouraging implications for organisations and policymakers: removing barriers and ensuring women have equal chances to participate in competitive endeavours could help narrow gender gaps, given that the ability to compete is essentially gender-equal in our context.

## 6. Conclusions

This study provides novel evidence that, within a Chinese context, women perform just as well as men in competitive tasks, and even outperform men when a risk element is introduced. These findings challenge the prevailing notion (based largely on Western data) that women are universally less effective in competitive environments. Instead, our results highlight the critical role of cultural context in shaping competitive outcomes. Understanding gender dynamics in competition in China is not only academically important but also practically relevant as Chinese women continue to rise in education and the workforce. Our evidence suggests that women’s competitive ability is unlikely to be a major barrier to their advancement; thus, efforts to close gender gaps should focus on other areas (such as encouraging women’s entry into competitions and addressing structural biases). Overall, our study challenges assumptions derived from Western-centric research and underscores the importance of examining cultural nuances when evaluating gender dynamics in competition and contributes to a more comprehensive understanding of these dynamics in the Chinese context. As China continues to play a larger role in the global economy, understanding how gender dynamics unfold in its labour force will be increasingly important for informing both local policy and international organisational practices.

## Figures and Tables

**Table 1:** Summary of participant characteristics.

		Observation (%) for Categorical Variables		
Variable		Total	Woman	Man
		(n = 326)	(n = 239)	(n = 87)
Top 20 Quintile	G1	16.9	15.1	21.8
	G2	13.5	13.0	14.9
	G3	13.8	14.2	12.6
	G4	10.1	10.5	9.2
	G5	17.2	15.9	20.7
Bottom 20 Quintile	G1	18.4	18.4	18.4
	G2	17.2	20.1	9.2
	G3	11.7	8.8	19.5
	G4	18.4	17.6	20.7
	G5	11.0	10.0	13.8
Level of Education	Undergraduate	41.6	48.5	23.3
	PG Taught	44.2	37.7	61.6
	PG Research	14.2	13.9	15.1
Academic Subjects	Accounting	21.8	24.7	14.0
	Finance	58.7	51.9	76.7
	International Economics and Trade	7.3	8.7	3.5
	International Trade	9.5	11.3	4.7
	Others	2.8	3.5	1.2
High Career Aspirations	Yes	53.6	56.7	45.3
	No	46.4	43.3	54.7
Confidence	Low	72.9	73.6	70.9
	High	27.1	26.4	29.1
		Mean (SD), Min, Max for continuous variables		
G1	Mean	6.9	6.8	7.3
	(sd)	2.8	2.7	2.8
	Min	0	0	0
	Max	13	13	13
G2	Mean	6.3	6.2	6.6
	(sd)	2.0	2.1	2.0
	Min	0	0	2
	Max	12	12	12
G3	Mean	10.3	10.6	9.2
	(sd)	4.6	4.2	5.5
	Min	−8	−2	−8
	Max	22	20	22
G4	Mean	20.7	20.9	20.2
	(sd)	10.2	9.7	11.4
	Min	−5	−5	−5
	Max	60	45	60
G5	Mean	5.3	5.3	5.2
	(sd)	2.3	2.2	2.6
	Min	0	0	0
	Max	12	12	12
Age	Mean	21.5	21.1	22.7
	(sd)	2.5	2.4	2.4
	Min	18	18	18
	Max	32	32	28

**Table 2:** Effect of gender on standardised scores and score quintiles in G1 and G5.

	(1)	(2)	(3)	(4)	(5)	(6)
Variables	Std. Score	Top 20	Bottom 20	Std. Score	Top 20	Bottom 20
Woman	−0.17	−0.06	0.00	0.07	−0.05	−0.04
	(0.13)	(0.04)	(0.05)	(0.14)	(0.05)	(0.04)
Constant	0.12			−0.05		
	(0.11)			(0.12)		
Observations	326	326	326	326	326	326
R-squared	0.01			0.00		

Standard errors in parentheses. * *p* < 0.10, ** *p* < 0.05, *** *p* < 0.01. Note: Models (1) and (4) employ linear regression models to examine relationships with standardised scores. Models (2), (3), (5), and (6) utilise probit models to analyse the probabilities of being in the top 20% and bottom 20%, and these models provide insights into the marginal effects (dy/dx).

**Table 3:** Effect of gender and incentives on standardised scores and score quintiles in all the games.

	(1)	(2)	(3)	(4)	(5)	(6)	(7)	(8)	(9)
Variables	Std. Score			Top 20			Bottom 20		
Woman	−0.17	−0.28 **		0.05	0.04		−0.02	0.02	
	(0.13)	(0.13)		(0.05)	(0.05)		(0.05)	(0.05)	
G2	0.02	0.00	0.00	0.00	0.00	0.00	−0.00	−0.00	0.00
	(0.15)	(0.15)	(0.13)	(0.06)	(0.06)	(0.00)	(0.06)	(0.06)	(0.00)
G3	−0.35 **	−0.39 **	−0.39 ***	−0.00	0.00	0.00	−0.00	−0.00	0.00
	(0.17)	(0.17)	(0.13)	(0.06)	(0.06)	(0.00)	(0.06)	(0.06)	(0.00)
G4	−0.17	−0.19	−0.19	0.00	0.00	0.00	−0.00	−0.00	0.00
	(0.16)	(0.16)	(0.13)	(0.06)	(0.06)	(0.00)	(0.06)	(0.06)	(0.00)
G5	−0.17	−0.22	−0.22 *	0.00	0.00	0.00	−0.00	−0.00	0.00
	(0.17)	(0.17)	(0.13)	(0.06)	(0.06)	(0.00)	(0.06)	(0.06)	(0.00)
Woman × G2	−0.03	−0.01	−0.01	−0.00	−0.00	0.00	0.00	0.00	0.00
	(0.18)	(0.18)	(0.15)	(0.07)	(0.07)	(0.00)	(0.07)	(0.07)	(0.00)
Woman × G3	0.47 **	0.51 ***	0.51 ***	0.00	−0.00	0.00	−0.00	0.00	0.00
	(0.19)	(0.19)	(0.15)	(0.07)	(0.07)	(0.00)	(0.07)	(0.07)	(0.00)
Woman × G4	0.23	0.25	0.25	−0.00	−0.00	0.00	−0.00	0.00	0.00
	(0.19)	(0.18)	(0.15)	(0.07)	(0.07)	(0.00)	(0.07)	(0.07)	(0.00)
Woman × G5	0.24	0.28	0.28 *	−0.00	−0.00	0.00	−0.00	0.00	0.00
	(0.19)	(0.19)	(0.15)	(0.07)	(0.07)	(0.00)	(0.07)	(0.07)	(0.00)
Controls		Yes	Yes		Yes	Yes		Yes	Yes
Fixed Effects			Yes			Yes			Yes
Constant	0.12	0.46	0.01			0.20			0.19
	(0.11)	(0.49)	(0.05)			(0.00)			(0.00)
Observations	1630	1585	1585	1630	1585	1585	1630	1585	1585
R-squared	0.01	0.04	0.01						

Notes: Standard errors in parentheses, * *p* < 0.10, ** *p* < 0.05, *** *p* < 0.01. Models (1) and (2) employ linear regression models to examine relationships with standardised scores. Models (4), (5), (7), and (8) utilise probit models to analyse the probabilities of being in the top 20% and bottom 20%, and these models provide insights into the marginal effects (dy/dx). Models (3), (6) and (9) are fixed effects models.

**Table 4:** Effect of gender, incentives, and confidence level on standardised scores and score quintiles.

	(1)	(2)	(3)	(4)	(5)	(6)	(7)	(8)	(9)
Variables	Std. Score			Top 20			Bottom 20		
Woman	−0.13	−0.23		0.17 **	0.17 **		−0.02	0.01	
	(0.14)	(0.14)		(0.08)	(0.07)		(0.05)	(0.05)	
G2	−0.07	−0.10	−0.10	−0.00	−0.00	0.00	0.00	0.00	0.00
	(0.17)	(0.16)	(0.16)	(0.10)	(0.10)	(0.00)	(0.06)	(0.06)	(0.00)
G3	−0.38 **	−0.44 **	−0.44 ***	−0.00	−0.00	0.00	0.00	0.00	0.00
	(0.19)	(0.19)	(0.16)	(0.10)	(0.10)	(0.00)	(0.06)	(0.06)	(0.00)
G4	−0.31 *	−0.34 **	−0.34 **	−0.00	−0.00	0.00	0.00	0.00	0.00
	(0.17)	(0.17)	(0.16)	(0.10)	(0.10)	(0.00)	(0.06)	(0.06)	(0.00)
G5	−0.20	−0.27	−0.27 *	0.00	−0.00	0.00	0.00	0.00	0.00
	(0.19)	(0.19)	(0.16)	(0.10)	(0.10)	(0.00)	(0.06)	(0.06)	(0.00)
High Confidence	0.44 *	0.39		0.39 ***	0.39 ***		−0.18 *	−0.17 *	
	(0.25)	(0.25)		(0.09)	(0.09)		(0.10)	(0.10)	
Woman × G2	0.08	0.12	0.12	0.00	0.00	0.00	−0.00	−0.00	0.00
	(0.20)	(0.19)	(0.18)	(0.11)	(0.10)	(0.00)	(0.07)	(0.08)	(0.00)
Woman × G3	0.51 **	0.57 ***	0.57 ***	0.00	0.00	0.00	−0.00	−0.00	0.00
	(0.22)	(0.22)	(0.18)	(0.11)	(0.10)	(0.00)	(0.07)	(0.08)	(0.00)
Woman × G4	0.35 *	0.37 *	0.37 **	0.00	0.00	0.00	−0.00	−0.00	0.00
	(0.20)	(0.20)	(0.18)	(0.11)	(0.10)	(0.00)	(0.07)	(0.08)	(0.00)
Woman × G5	0.19	0.26	0.26	−0.00	0.00	0.00	−0.00	−0.00	0.00
	(0.21)	(0.21)	(0.18)	(0.11)	(0.10)	(0.00)	(0.07)	(0.08)	(0.00)
Woman × High Confidence	−0.11	−0.10		−0.24 **	−0.25 **		0.01	0.03	
	(0.29)	(0.29)		(0.11)	(0.11)		(0.12)	(0.12)	
High Confidence × G2	0.32	0.35	0.35	0.00	0.00	0.00	−0.00	0.00	0.00
	(0.34)	(0.34)	(0.29)	(0.13)	(0.13)	(0.00)	(0.15)	(0.14)	(0.00)
High Confidence × G3	0.11	0.17	0.17	0.00	0.00	0.00	0.00	−0.00	0.00
	(0.38)	(0.37)	(0.29)	(0.13)	(0.13)	(0.00)	(0.15)	(0.14)	(0.00)
High Confidence × G4	0.48	0.51	0.51 *	0.00	0.00	0.00	0.00	−0.00	0.00
	(0.38)	(0.37)	(0.29)	(0.13)	(0.13)	(0.00)	(0.15)	(0.14)	(0.00)
High Confidence × G5	0.09	0.16	0.16	0.00	0.00	0.00	−0.00	−0.00	0.00
	(0.38)	(0.37)	(0.29)	(0.13)	(0.13)	(0.00)	(0.15)	(0.14)	(0.00)
Woman × High Confidence × G2	−0.39	−0.44	−0.44	−0.00	−0.00	0.00	0.00	−0.00	0.00
	(0.40)	(0.40)	(0.34)	(0.15)	(0.15)	(0.00)	(0.17)	(0.17)	(0.00)
Woman × High Confidence × G3	−0.15	−0.20	−0.20	−0.00	−0.00	0.00	0.00	0.00	0.00
	(0.43)	(0.43)	(0.34)	(0.15)	(0.15)	(0.00)	(0.17)	(0.17)	(0.00)
Woman × High Confidence × G4	−0.40	−0.43	−0.43	−0.00	−0.00	0.00	0.00	0.00	0.00
	(0.42)	(0.43)	(0.34)	(0.15)	(0.15)	(0.00)	(0.17)	(0.17)	(0.00)
Woman × High Confidence × G5	0.17	0.12	0.12	−0.00	−0.00	0.00	−0.00	−0.00	0.00
	(0.42)	(0.42)	(0.34)	(0.15)	(0.15)	(0.00)	(0.17)	(0.17)	(0.00)
Controls		Yes	Yes		Yes	Yes		Yes	Yes
Fixed Effects			Yes			Yes			Yes
Observations	1630	1585	1585	1630	1585	1585	1630	1585	1585
R-squared	0.05	0.08	0.02						

Standard errors in parentheses. * *p* < 0.10, ** *p* < 0.05, *** *p* < 0.01. Note: Models (1) and (2) employ linear regression models to examine relationships with standardised scores. Models (4), (5), (7), and (8) utilise probit models to analyse the probabilities of being in the top 20% and bottom 20%, and these models provide insights into the marginal effects (dy/dx). Models (3), (6) and (9) are fixed-effects models.

**Table 5:** Effect of gender, incentives, and career aspirations on standardised scores and score quintiles.

	(1)	(2)	(3)	(4)	(5)	(6)	(7)	(8)	(9)
Variables	Std. Score			Top 20			Bottom 20		
Woman	−0.25	−0.34 *		0.04	0.02		−0.01	0.02	
	(0.18)	(0.18)		(0.08)	(0.08)		(0.07)	(0.07)	
G2	−0.09	−0.09	−0.09	−0.00	0.00	0.00	−0.00	0.00	0.00
	(0.22)	(0.22)	(0.19)	(0.10)	(0.10)	(0.00)	(0.09)	(0.08)	(0.00)
G3	−0.50 *	−0.50 *	−0.50 **	−0.00	−0.00	0.00	−0.00	0.00	0.00
	(0.26)	(0.26)	(0.19)	(0.10)	(0.10)	(0.00)	(0.09)	(0.08)	(0.00)
G4	−0.21	−0.21	−0.21	−0.00	0.00	0.00	−0.00	0.00	0.00
	(0.23)	(0.23)	(0.19)	(0.10)	(0.10)	(0.00)	(0.09)	(0.08)	(0.00)
G5	−0.25	−0.25	−0.25	−0.00	0.00	0.00	−0.00	0.00	0.00
	(0.26)	(0.26)	(0.19)	(0.10)	(0.10)	(0.00)	(0.09)	(0.08)	(0.00)
High Career Aspiration	−0.14	−0.10		0.07	0.06		−0.01	−0.02	
	(0.22)	(0.22)		(0.09)	(0.09)		(0.08)	(0.08)	
Woman × G2	0.06	0.05	0.05	0.00	−0.00	0.00	0.00	−0.00	0.00
	(0.25)	(0.25)	(0.22)	(0.11)	(0.11)	(0.00)	(0.10)	(0.09)	(0.00)
Woman × G3	0.59 **	0.59 **	0.59 ***	0.00	0.00	0.00	0.00	−0.00	0.00
	(0.28)	(0.28)	(0.22)	(0.11)	(0.11)	(0.00)	(0.10)	(0.09)	(0.00)
Woman × G4	0.14	0.14	0.14	0.00	−0.00	0.00	0.00	−0.00	0.00
	(0.26)	(0.26)	(0.22)	(0.11)	(0.11)	(0.00)	(0.10)	(0.09)	(0.00)
Woman × G5	0.22	0.21	0.21	0.00	−0.00	0.00	0.00	−0.00	0.00
	(0.28)	(0.28)	(0.22)	(0.11)	(0.11)	(0.00)	(0.10)	(0.09)	(0.00)
Woman × High Career Aspiration	0.15	0.09		0.04	0.05		−0.03	−0.01	
	(0.26)	(0.25)		(0.10)	(0.10)		(0.10)	(0.10)	
High Career Aspiration × G2	0.21	0.17	0.17	0.00	−0.00	0.00	0.00	−0.00	0.00
	(0.31)	(0.30)	(0.26)	(0.13)	(0.13)	(0.00)	(0.12)	(0.12)	(0.00)
High Career Aspiration × G3	0.26	0.20	0.20	0.00	−0.00	0.00	0.00	−0.00	0.00
	(0.34)	(0.34)	(0.26)	(0.13)	(0.13)	(0.00)	(0.12)	(0.12)	(0.00)
High Career Aspiration × G4	0.05	0.03	0.03	0.00	−0.00	0.00	0.00	−0.00	0.00
	(0.33)	(0.32)	(0.26)	(0.13)	(0.13)	(0.00)	(0.12)	(0.12)	(0.00)
High Career Aspiration × G5	0.13	0.06	0.06	0.00	−0.00	0.00	0.00	−0.00	0.00
	(0.34)	(0.34)	(0.26)	(0.13)	(0.13)	(0.00)	(0.12)	(0.12)	(0.00)
Woman × High Career Aspiration × G2	−0.14	−0.10	−0.10	−0.00	0.00	0.00	−0.00	0.00	0.00
	(0.36)	(0.36)	(0.31)	(0.15)	(0.15)	(0.00)	(0.14)	(0.14)	(0.00)
Woman × High Career Aspiration × G3	−0.16	−0.10	−0.10	−0.00	−0.00	0.00	−0.00	0.00	0.00
	(0.39)	(0.38)	(0.31)	(0.15)	(0.15)	(0.00)	(0.14)	(0.14)	(0.00)
Woman × High Career Aspiration × G4	0.25	0.27	0.27	−0.00	0.00	0.00	−0.00	0.00	0.00
	(0.38)	(0.37)	(0.31)	(0.15)	(0.15)	(0.00)	(0.14)	(0.14)	(0.00)
Woman × High Career Aspiration × G5	0.08	0.17	0.17	0.00	0.00	0.00	−0.00	0.00	0.00
	(0.38)	(0.38)	(0.31)	(0.15)	(0.15)	(0.00)	(0.14)	(0.14)	(0.00)
Controls		Yes	Yes		Yes	Yes		Yes	Yes
Fixed Effects			Yes			Yes			Yes
Constant	0.20	0.59	0.01			0.20			0.18
	(0.16)	(0.51)	(0.05)			(0.00)			(0.00)
Observations	1610	1580	1580	1610	1580	1580	1610	1580	1580
R-squared	0.01	0.04	0.02						

Standard errors in parentheses. * *p* < 0.10, ** *p* < 0.05, *** *p* < 0.01. Note: Models (1) and (2) employ linear regression models to examine relationships with standardised scores. Models (4), (5), (7), and (8) utilise probit models to analyse the probabilities of being in the top 20% and bottom 20%, and these models provide insights into the marginal effects (dy/dx). Models (3), (6) and (9) are fixed-effects models.

## Data Availability

The data that has been used is confidential.
